# Access to Resources in the Community Through Navigation: Protocol for a Mixed-Methods Feasibility Study

**DOI:** 10.2196/11022

**Published:** 2019-01-24

**Authors:** Simone Dahrouge, Alain Gauthier, Francois Chiocchio, Justin Presseau, Claire Kendall, Manon Lemonde, Marie-Hélène Chomienne, Andrea Perna, Darene Toal-Sullivan, Rose A Devlin, Patrick Timony, Denis Prud'homme

**Affiliations:** 1 Bruyere Research Institute Department of Family Medicine University of Ottawa Ottawa, ON Canada; 2 Centre for Rural and Northern Health Research Laurentian University Sudbury, ON Canada; 3 Telfer School of Management University of Ottawa Ottawa, ON Canada; 4 Clinical Epidemiology Program The Ottawa Hospital Ottawa, ON Canada; 5 University of Ontario Institute of Technology Oshawa, ON Canada; 6 Institut du Savoir Montfort Ottawa, ON Canada; 7 Bruyere Research Institute Ottawa, ON Canada; 8 University of Ottawa Ottawa, ON Canada

**Keywords:** access, community resources, feasibility study, navigation, primary health care

## Abstract

**Background:**

Community-based health and social resources can help individuals with complex health and social needs achieve their health goals. However, there is often inadequate access to these resources due to a lack of physician and patient awareness of available resources and the presence of social barriers that limit an individual’s ability to reach these services. Navigation services, where a person is tasked with helping connect patients to community resources, embedded within primary care may facilitate access and strengthen the continuity of care for patients.

**Objective:**

This study aims to describe the protocol to assess whether the implementation of the Access to Resources in the Community (ARC) navigation model (an innovative approach to navigation services) is feasible, including its potential to achieve its intended outcomes, and to assess the viability of the evaluation approach.

**Methods:**

The study consists of a single-arm, prospective, explanatory, mixed-methods, pre-post design feasibility study focusing on primary care practice settings with vulnerable populations. Participants include primary care providers and patients.

**Results:**

Enrollment is closed with 82 patients. Navigation services have ended for 69 patients.

**Conclusions:**

The study of an innovative complex intervention requires an adequate assessment of the feasibility of the intended approach during which the potential challenges of the planned intervention and need for its adaptation may be uncovered. Undertaking a feasibility study of the ARC navigation model from a conceptually clear and methodologically solid protocol will inform on the practicality and acceptability of the approach, demand for the services, ease of implementation, quality of integration of the new services within primary care, and practicality and potential for efficacy prior to initiating a randomized controlled trial.

**Trial Registration:**

ClinicalTrials.gov NCT03105635; https://clinicaltrials.gov/ct2/show/NCT03105635 (Archived by WebCite at hhttp://www.webcitation.org/75FrwXORl)

**International Registered Report Identifier (IRRID):**

RR1-10.2196/11022

## Introduction

Equitable access to primary health care (PHC) plays an important role in reducing health inequities [1]. Despite considerable efforts to strengthen that sector in Canada [2], disparities in access to these services continue to affect several populations, including immigrants, indigenous people, individuals of lower socioeconomic status, those living in rural regions, and cultural minorities, including Francophones living in minority situations [3-9]. This problem is compounded by the fact that these social factors are also determinants of health that contribute markedly to the risk of poor health [10] and adequate access to PHC, including health-enabling resources available in the community, which remains a priority strategy to mitigating these inequities [11].

Community resources (CRs) such as smoking cessation, falls prevention, and chronic disease self-management programs can play an important role in supporting individuals achieve their health goals. Primary care providers (PCPs) may offer lifestyle counseling and preventive care support to promote positive health behaviors, but this is often insufficient for individuals to meet their intended goals because the path to healthy behavior is fraught with barriers that thwart their intent and capacity to act [12]. The work done by PCPs in supporting individuals to develop self-efficacy in the management of their health can be complemented by accessing health-enabling CRs, which encompass a broad range of health and social services. Several reviews report positive outcomes with community-based services that aim to reduce the risk of cardiovascular disease [13-15], promote secondary prevention of various chronic diseases [16-18], and improve health-promoting behaviors [19,20]. Such resources are recommended by the US Preventive Services Task Force [21] and the American Heart Association [19]. Similarly, National Guidelines for Diabetes Management highlight the role self-management education and community support programs play in achieving healthy outcomes [22]. Many of these resources have been shown to be useful in meeting the health needs of individuals with complex social profiles [15,23] and some have highlighted their role in reducing health inequities experienced by certain populations [24,25]. Unfortunately, these services remain underused [26], especially by individuals with social disadvantages [27], resulting in the propagation of inequities and unmet health needs.

One response to this multifaceted issue is to facilitate access to health-enabling CRs by embedding navigation services within PHC. Navigators may be nonclinical individuals or health professionals who assist patients in identifying the appropriate CRs and support them in overcoming access barriers and achieving service utilization [28]. However, the navigator role has been almost exclusively studied in disease- or population-specific contexts [29,30]. In the latter role, they are often called community health workers. Two recent meta- analyses focused on medical conditions, the majority of which were cancer-related studies, showed that patients assigned to navigation services exhibited markedly better outcomes across a number of measures, including appropriate health care utilization, disease control, and clinical outcomes, such as mortality [29]. Another review reported similar benefits of navigation services for immigrants and ethnic communities [31].

The current models of navigation services and their implementation have considerable limitations. Models targeting individuals with specific medical conditions do not address the breadth of potential navigation needs individuals may have and may be contributing to a fragmented delivery of care. Population-specific programs only target a subset of the population and, by definition, cannot be applied to the general population. Implementing changes in the way PCPs operate— even when there is agreement within PCPs on the need for the change—is challenging as well. To date, a very few studies have implemented navigation services within primary care, integrating these services within the breadth of care they coordinate [32-37] and supporting the PCPs’ efforts to engage patients in self-care. We found a single study that evaluated the role of a patient navigator in providing system navigation support to more complex primary care patients; however, in that study, the navigation services were provided by a social worker [38].

Informed by evidence and in consultation with key stakeholders, we developed a novel approach intended to enhance equitable Access to Resources in the Community (ARC)—the ARC model. The ARC model consists of implementing small changes to primary care practices that would encourage PCPs to direct patients to resources in the community that could help them address their health and well-being needs. In parallel, a nonclinical navigator attached to the practice would support these patients tot identify the most appropriate service and overcome barriers that might prevent them from making use of the recommended resources. This feasibility study will assess whether the ARC model is feasible, including its potential to achieve its intended outcomes, and the viability of the evaluation approach. Ultimately, this feasibility study will strengthen a subsequent randomized controlled trial, which, in turn, will increase the likelihood of collecting reliable and relevant data and produce valid conclusions on the implementation and impact of our navigator program.

Specifically, we will evaluate 8 areas of feasibility: Acceptability, Demand, Implementation, Adaptation, Integration, Practicality, Efficacy of the ARC model, and Appropriateness of the intervention evaluation approach to study participants [39].

## Methods

### Reporting and Design

We have followed the Standard Protocol Items: Recommendations for Interventional Trials guidelines for reporting on protocols [40]. This is a single-arm, prospective, explanatory, mixed-methods, pre-post design feasibility study.

### Setting

The study is set in central Ottawa (Ontario, Canada), a region with broad socioeconomic diversity, including Francophones (19%), immigrants (19%), visible minorities (18%), and seniors (16%) [41].

### Participants

The ARC model was implemented in 2 practice contexts to understand the different levers and barriers to implementing the ARC model: (1) the traditional primary care practice, which consists of family physicians, nurses, and administrative staff; and (2) the interprofessional practice model in which patients also have access to various allied health professionals such as nurse practitioners, pharmacists, and social workers, as well as some in-house health programs. Participating PCPs are required to provide comprehensive primary care services to the general population.

Patients of participating PCPs were eligible to participate if one or more needs, which may be addressed by services offered in the community, are identified during an encounter with their PCP, they are able to communicate in English or French or willing to be served by a cultural interpreter or translator, and have no marked cognitive deficiencies or have a family member that can provide proxy consent and participate in the study with the patient.

### Ethics Approval

This study was approved by the following ethics boards: Ottawa Health Science Network Research Ethics Board (#20160914-01H), Bruyère Continuing Care Research Ethics Board (#M16-16-055), University of Ottawa Research Ethics Board (#A05-17-04), and L’Hôpital Montfort Research Ethics Board (#SD-DP-27-02-17).

### Intervention

We established a Collaborative Partnership of key stakeholders to inform the development, implementation, and evaluation of the ARC navigation model. The partnership includes policy makers, members from community organizations, health care providers, health planners, and people with lived experience of health care as a consumer or caregiver. The ARC intervention intends to promote equitable access to health-enabling resources by engaging PCPs in identifying their patients’ needs that could be addressed by a CR and directing them to such services. Patients’ access to these resources is subsequently supported through navigation. A logic model (presented in Multimedia Appendix 1) was developed to establish the expected links among the intervention components, planned activities, and anticipated outcomes. The ARC navigation model is presented in Multimedia Appendix 2, and details of the ARC intervention are described in Multimedia Appendix 3 (Template for Intervention Description and Replication Checklist [42]). The study promotional materials for patients are offered in Multimedia Appendices 4 and 5 and the ARC referral form and instructional video for PCPs are presented in Multimedia Appendices 6 and 7. The two main intervention components are (1) changes to the practice environment to enhance PCP recommendations for patients to access CRs that could address their needs; and (2) navigation support for these patients to help them achieve access to resources. Briefly, we relied on Rogers’ Diffusion of Innovation theory [43] to underpin the practice changes required to promote referrals to CRs in primary care practices. These changes were informed by a rapid realist review conducted by us to understand the most likely effective approaches and their potential benefit in our context [44]. The navigator’s role was founded on the Health Action Process Approach [45] and aims to support patients in achieving their health and wellness goals by promoting access to health-enabling resources in the community.

The role of the navigator is limited to nonclinical activities that support access to community services that address a wide range of health and social needs. Multiple sources were used to inform the role of the navigator including (1) peer-reviewed literature about the scope of navigation activities provided with different populations, medical conditions, and contexts [38,46-49]; (2) best practice guidelines for implementing and evaluating community health worker programs [37]; and (3) literature on navigation training programs.

The ARC navigator is a lay person with no clinical background who is hired by the research team and trained to provide navigation services. PCPs were encouraged to see that new team member as someone lending a helping hand to patients in need of support to gain access to needed services—the type of support a well-informed family member could potentially provide. It was made clear that the navigator is a lay individual and would not be expected to address any clinical issues and that should such issues arise, they would be communicated back to the PCPs for their action. We sought to hire an individual with excellent communication skills, evident empathic qualities, and good management abilities. The navigator training program is based on competencies developed specifically for their role in primary care. The key competencies are to provide basic navigation services and identify appropriate resources within our context, demonstrate effective interpersonal communication including cultural and linguistic sensitivity, collaborate and work effectively with primary care team and CR program staff, advocate for patients and intervene with services to promote access to needed resources, demonstrate commitment to professional responsibilities and ongoing learning, and educate and empower patients about CRs for their health.

The navigator would spend a minimum of one half day at the practice during which they would meet with patients referred to them and interact with the members of the primary care team. The navigator did not chart in the patient medical records. Written communication between the navigator and primary care team was by fax. The expectation was that PCPs, sensitized to the availability of CRs that could complement the care they provide to their patients and confident that more socially complex patients can receive the assistance they require to support access to these resources (from the navigator), would be more likely to identify patient needs that could be addressed by CRs, discuss these during their encounter, and if appropriate, make the recommendation to access these services. The navigator would then meet with each patient to whom a service was recommended to understand their needs, expectations and priorities, identify anticipated barriers in accessing the resource (eg, knowledge, health literacy, transportation, completing forms, caregiving responsibilities, financial, and motivation), support patients in overcoming these barriers, and facilitate access to the most appropriate resource for patients. In addition, the navigator was trained to use communication strategies, such as motivational interviewing [50], that would encourage engagement of patients in identifying what is important to them, what they perceive as challenges in accessing CRs, and the navigation activities needed. Patient self-efficacy is fostered through providing various forms of social support including providing information, emotional support, and guidance or accompaniment to access CRs. The navigator would report back to the PCP on the plan developed after the first encounter and again at the end of services. Navigation services would be discontinued when a patient has accessed the appropriate service or no longer wants or requires navigation assistance. Navigation services are offered to patients for up to 3 months.

### Timelines

The patient recruitment period was 9 months, and individual patient participation will be approximately 3 months.

### Sample Size

Sample sizes of 30 [51] or 50 [52] are commonly recommended for feasibility studies. We based our sample size on the ability to adequately assess the demand for the navigation services and to estimate the potential for patients to achieve the intended access to the resource. We estimated that the participation of 4-6 practices, each with at least 3 PCPs (minimum 12 PCPs) caring for a panel size of 1500 patients, would be required. We assumed conservatively that 30% of patients at the practice could potentially benefit from a CR. In the region where the study is being conducted, >60% of the population has insufficient consumption of fruits and vegetables and 28% individuals are obese, 19% drink heavily, 12% smoke, and 22% report feeling stressed “quite a lot” [53]. The 95% CI range for these outcome measures would be <3% for referral rate (5400 potentially eligible individuals), <15% for participation rate (if conservatively 162/5400, 3.00%, are referred), and <22% for the access measure (assuming a participation rate of 81/162, 50.0%). We also aimed to interview a minimum of 1 PCP and 2 patients per practice.

### Recruitment

The study was promoted among stakeholders. Providers expressing interest were sent a study information and consent form, and a recruitment session was scheduled with all interested PCPs at the practice.

During encounters with patients, when participating PCPs and their patients identified a need that could be addressed by a CR, the PCP completed a standardized CR referral form, briefly introduced the study, and requested the patient’s agreement to be contacted by a member of the research team. Patients who agreed to be contacted received a study information and consent package and a copy of the completed referral form identifying their need(s). A copy of that form was faxed to the study team who then contacted the patient and provided detailed information about the study. Patients who provided verbal consent to participate in the study were asked to sign and mail in the consent form included in their study information and consent package.

A subsample of PCPs and patients participated in the qualitative component of the study, which consisted of an interview at the beginning and again at the end of their participation in the study. All PCPs were invited to participate in the interviews, with the aim to enroll at least 1 PCP per practice. Patients were purposefully selected to be invited for the interviews based on their responses to the baseline survey. The recruitment aimed to maximize variation in social complexity and ensure variability in age and gender. At least 1 patient from each practice was required.

### Data Collection Methods

Table 1 provides a summary of the data collection tools, the dimensions measured, the population targeted, as well as how and when the tools are to be administered.

One practice member, referred to as the practice champion, is the main contact for the practice. The practice champion completes the practice survey, and each participating PCP completes the provider survey. These surveys are completed at baseline before the introduction of the patient navigator in the practice, and again immediately prior to ending the navigation services. These surveys assess the practice’s organization and PCPs’ knowledge, attitudes, and experience with reference to vulnerable populations, as well as factors that can influence the success of the intervention and its implementation from a change management perspective, including the organizational structure [54], climate of their work environment [55], as well as readiness [56] and commitment to change [57]. Furthermore, the postintervention provider survey includes questions relating to their experience with the patient navigator.

Patients complete a preintervention survey immediately after providing consent and prior to meeting with the navigator, and a postintervention survey at 3 months. These surveys assess various dimensions of access, measures of self-efficacy, social vulnerability, and their experience with CRs and the ARC navigation services.

A subset of patients and PCPs are also invited to participate in an interview following the completion of the pre- and postintervention surveys. These interviews explore patients’ access to PHC services and providers’ experience providing care to vulnerable patient populations. In addition, patients and PCPs are asked about their experience with the patient navigator. These interviews will be used to understand and build upon the survey results [58].

**Table 1:** Data collection tools.

Instrument and dimension			Target population	Administration	
				How	When
**Quantitative data**					
	Referral form: Patient needs; Referral rate		PCPs^a^, patients	Completed by PCPs in collaboration with patients	Completed for each referral throughout the study
	Practice survey^b^: Organization, services provided		1 per practice	Self-administered by 1 PCP per practice	Baseline and end of the study (1 month prior to end of patient recruitment)
	**Provider survey^b^**				
		Equity orientation; Climate; Organizational structure; Change readiness	All PCPs	Self-administered by PCPs	Baseline and end of the study
		Experience with the intervention	All PCPs	Self-administered by PCPs	End of the study (1 month prior to end of patient recruitment)
	**Patient survey**				
		Experience with health care, various dimensions of access, self-efficacy, social vulnerability, Health Action Process Approach, Patient Activation Measure, experience with community resources	All patients referred	Administered via telephone by the research team	Preintervention and 3 months postintervention
		Experience with intervention, utilization of recommended community resource	All patients referred	Administered via telephone by the research team	3 months postintervention
**Qualitative data**					
	**Provider interview**				
		Background (PCP and practice profile) and expectations	2 PCPs per practice	Administered in-person by the research team	Baseline
		Experience with intervention	2 PCPs per practice	Administered in-person by the research team	End of the study (after completing “end of the study” survey)
	**Patient interview**				
		Experience with health care access	2 patients per practice	Administered in-person or by phone by the research team	Preintervention
		Experience with intervention	2 patients per practice	Administered in-person or by phone by the research team	Postintervention
	Rapid cycle evaluation: Acceptability of intervention activities; Integration of study activities in the practice		1 per practice	Self-administered by 1 PCP per practice	Set-up of study activities (1-month implementation phase), bimonthly throughout the study
	Navigator interview: Training, capacity, challenges, suggestions for improvement		Navigator	Administered in-person by the research team	End of the study
**Study documentation**					
	Coordinator log: Encounters with practices; Weekly (or more) debriefs with Navigator		Study coordinator	Study coordinator	Throughout the study
	Navigator log: Encounters with PCPs, patients and community resources; Navigation process and activities; Navigator reflections		Patients who accepted Navigator services	Completed by the navigator	Throughout the study
	Ultra-observational tool: Practice environment		1 per practice	Research team	Baseline
	TIDieR^c^: Intervention delivery and fidelity		Overall	Research team	Throughout the study
	Stange and Glasgow Tool: Practice environment context, validity of the intervention		1 per practice	Research team	End of the study

^a^PCP: primary care provider.

^b^Practice and Provider surveys conducted at “end of study” are delivered 1 month prior to the end of patient recruitment. Provider interviews conducted at “end of the study” are performed after the provider survey has been received.

^c^TIDieR: Template for Intervention Description and Replication.

Conforming to a rapid cycle evaluation (RCE) approach, the designated practice champion completes regular assessments of the study progress to inform the need for adapting the intervention to meet the needs of the practice; this evaluation also informs on levers and barriers to the changes imposed by the introduction of an additional layer of services. The first evaluation was conducted immediately following the initial implementation of the intervention and assessed the practices’ experience with the introduction of the components of the ARC intervention. Subsequent evaluations were conducted bimonthly to evaluate their impressions of study progress [59].

Throughout the study, the coordinator maintains a log of activities relating to encounters with practices and weekly debriefs with the navigator. The patient navigator maintains a log of activities relating to patient support, including encounters with patients, their PCPs, and staff from the recommended CR. Furthermore, the patient navigator completes a reflective journal describing their thoughts related to day-to-day activities to promote deeper understanding of the knowledge and skills required to carry out their role.

### Outcomes

The feasibility study will assess 8 areas of focus: Acceptability of the ARC model; Demand for the navigation service; Implementation approach viability; Adaptation required; Integration of the navigation service within the practice; Practicality of the ARC model to the practice, providers, and patients; the potential for the intervention Efficacy; and Appropriateness of the intervention evaluation approach to study participants [39]. Table 2 provides the operational definition of the areas and their data source.

### Data Management

Procedures developed by the ARC team and captured in various training and “how to” guides contributed to the standardized implementation of study activities related to data collection, coding, entry, and storage. Quantitative data are inscribed directly into Qualtrics, a centralized data collection tool, and transferred to SPSS (IBM) for analyses. For qualitative data, interview notes or transcripts and open-ended answers to survey questions are entered into (NVivo; QSR International), a software that facilitates content analysis.

### Analyses

Table 2 provides a summary of the analytical approach to each of the outcome measures related to 8 areas of focus of a feasibility study. Consistent with principles of an explanatory sequential design of a phenomenological tradition, we will begin by analyzing the patient and provider surveys, the results of which will guide the qualitative line of questioning, which will seek to further explore survey findings. The overarching research question guiding this qualitative phase is, “What is the provider and patient experience with navigation to CRs?” Questions for the patient and provider interview guides align with elements of the study’s conceptual framework including Rogers’ Diffusion of Innovation theory [43], the Health Action Process Approach [45], and elements of the Access Framework [60]. The interview questions will be further developed on the basis of the quantitative results as they emerge, and coding and content analysis will follow a sequential iterative process [61,62]. The RCE data are analyzed qualitatively to inform the ongoing adaptation of the intervention integration within each practice. While the elements of the intervention were theory driven, how these are applied within complex systems, such as primary care practices, must be informed by that context. We anticipated that team composition, use of electronic medical records tools, and clinic layout and flow would be influential factors but expected that additional context factors would emerge through the RCE [43,63,64].

**Table 2:** Summary of feasibility outcome measures.

Area of focus	Outcome measure	Measurement tool	Analyses
Acceptability	PCP^a^ satisfaction with study activities PCP commitment to change PCP and patient experience with the Navigator	Rapid cycle evaluation (implementation stage) Post-PCP and patient surveys and interviews	Level of satisfaction with study activities Descriptive statistics Descriptive statistics and content analysis
Demand	Referral forms completed by PCPs Patient use of navigation services Navigator-patient encounters	Referral form Navigator log	Rate of referrals Proportion of patients using navigation services Number of navigator-patient encounters
Implementation	PCP readiness to change to accept the ARC navigation model Mode of delivery of navigation services	Pre- and post-PCP surveys Navigator Log	Descriptive statistics Number of telephone versus in-person encounters
Adaptation	Changes in the planned process to accommodate practices Changes in the method of navigation services delivery to accommodate patients’ expectations	Rapid cycle evaluation (intervention stage) Navigator log	Frequency of adaptation of study activities Proportion of phone versus in-person encounters Proportion of in-person encounters at the practice versus elsewhere
Integration	PCP satisfaction with study activities PCP satisfaction with intervention activities Appropriateness of navigator service delivery Navigator and PCP communication	Pre- and postpractice surveys Rapid cycle evaluation (intervention stage) Navigator log Rapid cycle evaluation (intervention stage) Post-PCP survey and interview	Comparison across practice models Descriptive statistics and content analysis Frequency of patient-navigator encounters at the practice site Descriptive statistics and content analysis
Practicality	PCPs’ ability to perform study activities Patient ability to use navigator services	Rapid cycle evaluation (intervention stage) Postpatient survey and interview	Descriptive statistics and content analysis
Efficacy	Ability of patients to access CR that meet their needs Characteristics of patients and needs according to ability to access CR	Postpatient survey and interview	Descriptive statistics and content analysis
Appropriateness of evaluation	Completeness of surveys (and individual components) by PCPs and patients and participants’ comments on these (eg, content, clarity, and length) Participation of PCP and patients in interviews	PCP and patient surveys and interviews	Proportion of surveys included Number of interviews completed

^a^PCP: primary care provider.

## Results

Participant recruitment has ended and data collection is still in progress. Overall, 35 PCPs consented to participate in the study, 29 of which referred at least 1 patient. Across the 9-month intervention period, 131 referrals were received by the research team. Patient enrollment is closed, with 82 patients participating out of a possible 131 patients (62.6% response rate). Of the 131 patients, the research team was unable to make direct contact with 34 (26.0%) patients and 15 (11.5%) patients declined to participate in the study. Of the 82 enrolled patients, 3 (4%) withdrew from the study after completing the baseline survey; 78 (99%) patients accepted navigation services, and 69 (87%) patients completed these services to date. Postintervention data collection is ongoing. Results informing the feasibility of the ARC navigation model according to the 8 areas of focus described above will be made available.

## Discussion

There is a need to implement measures that will foster better use of CRs, especially for vulnerable populations. There is also a need to assess the extent to which such measures actually meet their objectives. We identified navigation services attached to primary care as an innovative means by which patients’ trajectories from primary care practices to CRs can be facilitated. In addition, we recognize that both the need to implement navigation services in primary care and the need to assess their impact are complex conceptually and operationally. As such, it is sensible and logical to first assess the acceptability, implementation, integration, practicality, and potential adaptation of both the intervention and research process through a feasibility study. This feasibility study will strengthen a subsequent randomized controlled trial, which, in turn, will increase the likelihood of collecting reliable and relevant data and produce valid conclusions on the implementation and impact of our navigator program.

## Appendix

Multimedia Appendix 1 Logic Model.

Multimedia Appendix 2 The ARC Navigation Model.

Multimedia Appendix 3 Intervention (Based on TIDieR checklist).

Multimedia Appendix 4 ARC Promotional Poster.

Multimedia Appendix 5 ARC Promotional Video.

Multimedia Appendix 6 ARC Referral Form.

Multimedia Appendix 7 ARC Instructional Video.

## Abbreviations

ARC: Access to Resources in the Community
CR: community resource
PHC: primary health care
PCP: primary care provider
RCE: rapid cycle evaluation
TIDieR: Template for Intervention Description and Replication
