# Supplementary material for: Access to Resources in the Community Through Navigation: Protocol for a Mixed-Methods Feasibility Study
Source: JMIR Res Protoc. 2019 Jan 24;8(1):e11022. doi: 10.2196/11022 (PMC6365876; doi:10.2196/11022)
Supplement: Multimedia Appendix 1 [file resprot_v8i1e11022_app1.pdf]

# The ARC Logic Model

| Resources | Activities | Outputs | Short-Term Outcomes<br>(During study period = approx. one year) | Mid-term Outcomes<br>1-5 years | LTO<br>> 5 years |
|---|---|---|---|---|---|
| **HR:**<br>• Study Coordinator<br>• Research Assistant<br>• Providers & practice staff<br>• Patient Navigators<br><br>**Resources:**<br>• Champlain Healthline<br>• Ontario 211<br>• Community health and social services | **Implementation**<br>• **Practice**<br>- Facilitation activities to assess practice needs and preferences, optimize referral process based on practice preference<br>- Orientation on community resources<br>- Establish patient promotional material<br>- Establish navigator implementation adapted for each practice<br>- Adapt implementation based on rapid cycle evaluation<br><br>• **Navigator**<br>- Train navigators and attach to PC practices<br>**Ongoing**<br>- Navigators assess patients' needs and provide support to patients to help reach resources | **Implementation**<br>• **Practice**<br>- # PC practice training sessions held<br>- PC practice training session attendance<br>-Referral process adapted & implemented<br><br>• **Navigator**<br>- Education package/modules<br>- # Navigators recruited, trained<br>- Navigator competence<br><br>- # Navigators days (hours)/practices<br>- # Patients/navigator: patient profile, type of resources, types of activities,…<br>- List of services with updated info<br>- # Communication with provider<br><br>**Ongoing**<br>• **Practice**<br>- # promotional info taken<br><br>• **Navigator** | • **Providers**<br>- Increased awareness of CR<br>- Increased confidence and motivation to refer to CR<br>- Improve providers comfort in caring for vulnerable populations<br><br>- Increased referrals<br>- Increased referrals to vulnerable/French population<br><br>- Acceptability of practice change<br>- Acceptability of navigator role/collaboration/ communication<br><br>• **Patients**<br>- Increased awareness of CR and languages in which they are available<br>- Increased knowledge of benefits of CR<br>- Increased confidence and motivation to access CR in preferred language<br><br>- Increased utilization of CR<br>- Reduced number of barriers to access CR<br>- Increased equitable utilization of CR across social strata<br><br>- Improved self-efficacy (navigation and health management)<br>- Reduced unmet health needs<br><br>- Acceptability of navigator role and encounters | • **Practices**<br>- Sustainability of model (referral, navigator)<br><br>• **Patients**<br>- Reduced risk of poor health outcome related to benefit from accessing CR (Reduced ER/Hospitalization)<br>- Improved system efficiency (Reduced visits to PCP)<br>- Reduced health inequities across social strata<br><br>• **System**<br>- Improved availability of appropriate CR<br>- Improved integration and cohesion of services | Improved population health and well-being |

| | | | | | |
|---|---|---|---|---|---|
| | | - # Navigator days (hours)/practices<br>- # Patients/navigator by patient profile<br>-# Patient encounters with navigators<br>-Navigator activities (type of resources, type of support activities)<br>-Type of patient barriers addressed<br>-List of services with updated info<br>-# times communicated with provider<br>-Confident/competent in their tasks | - Perception of information continuity across navigator and PCP<br><br>• **System**<br>-Knowledge of gaps in availability of services in appropriate language<br>-Knowledge of patient experience in accessing CR | | |
