# Supplementary material for: Access to Resources in the Community Through Navigation: Protocol for a Mixed-Methods Feasibility Study
Source: JMIR Res Protoc. 2019 Jan 24;8(1):e11022. doi: 10.2196/11022 (PMC6365876; doi:10.2196/11022)
Supplement: Multimedia Appendix 2 [file resprot_v8i1e11022_app2.pdf]

The ARC Navigation Model

The ARC navigation model is intended to address gaps in the existing navigation models. This model of navigation is quite distinct from existing models in the following ways:

- **The navigation services support access to community services**
  The ARC navigator connects patients to community services that address diseases prevention and health promotion, whereas existing navigation services do not.
- **The navigator is attached to primary care practices.**
  The ARC navigator is a member of the primary care team and promotes integration of primary and community care by 1. Improving continuity of information, and 2. Fostering recommendations to community resources by enabling patients' access to these resources.
- **The navigator is a "generalist".**
  The ARC navigator supports a broad patient population (e.g., a general population not characterized by a specific cultural background or illness) and promotes access to resources that address a wide range of needs.
- **The navigator is a lay person**
  The ARC navigator is a lay person with no clinical background or medical training. The navigator is trained specifically in navigation service delivery and so has a better knowledge of the context and existing resources.
- **The navigator is linked to a Community Health Centre**
  A partnership with a local Community Health Centre enables the ARC navigator to be hosted within their premise and work alongside their multicultural health navigators.
