# Supplementary material for: Access to Resources in the Community Through Navigation: Protocol for a Mixed-Methods Feasibility Study
Source: JMIR Res Protoc. 2019 Jan 24;8(1):e11022. doi: 10.2196/11022 (PMC6365876; doi:10.2196/11022)
Supplement: Multimedia Appendix 3 [file resprot_v8i1e11022_app3.pdf]

The ARC Intervention (Based on TIDieR checklist)

A. WHAT

i. Materials:

1. Objective #1: Enhance recommendations to CR

1.1. Orientation on community resources

- A list of the various types of health and social resources that address patient priority needs identified by PCPs for each practice

1.2. Promotional material that highlights the breadth of available CR and provides information on the ARC study and the availability of the patient navigator

- ARC promotional poster (see Multimedia Appendix 4 – ARC Promotional Poster)
- ARC promotional video (see Multimedia Appendix 5 – ARC Promotional Video)

1.3. Referral process

- ARC referral form (see Multimedia Appendix 6 – ARC Referral Form)
- Participating PCPs will receive a face-to-face training on the referral process (e.g., how to complete the referral form and share a copy with the patient and the research team)
- An instructional video describing the referral process will be shared with all participating PCPs (see Multimedia Appendix 7 – ARC Instructional Video)
- A step-by-step guide outlining the steps of the ARC referral process will be shared with PCPs.

2. Objective #2: Enhance equitable utilization of CR

- Navigator training (online, face-to-face, community mentorship) developed by the research team (https://researchprogram.wixsite.com/arcproject)

ii. Procedures:

1. Enhance recommendations to CR

1.1. Orientation on community resources

- The research coordinator will meet with participating PCPs at each practice to provide an orientation on the availability of existing health and social resources in the community

1.2. Promotional material

- The ARC promotional poster and video (where feasible) will be displayed in the waiting room of participating practices, and encourage patients to discuss the potential benefit of CRs in addressing their needs.

1.3. Referral process

- The ARC referral form will be available on the practice EMR
- Providers will complete the ARC referral form on the EMR and fax a copy of the form to the research team.
- A copy of the referral form will be provided to the patient
- Approaches to streamlining the referral process will be tailored according to the needs and preferences of PCPs; the ARC referral form may subsequently be adapted within each practice to optimize the referral process

2. Enhance equitable utilization of CR

2.1. Support patients to overcome barriers to access

- A patient navigator will provide non-clinical navigation services to support patients identify and overcome barriers to accessing the CR to which they were referred.

2.2. Integrate patient navigator services into primary care practice

- PCPs will select the time(s) of week when the patient navigator will be present in the practice and identify the space and resources allocated to them on these days.
- The patient navigator will be available to see their assigned patients or have discussions with practice staff to clarify their roles and responsibilities.
- PCPs will specify their expectations for feedback on referrals, and select the preferred means of communication (e.g., receiving periodic reports, exchanging messages via EMR, etc.)

B. WHO PROVIDED

1. Enhance recommendations to CR

1.1. Orientation to community resources developed and delivered by the research team

1.2. Promotional material on community resources and the ARC study developed by the research team in collaboration with advisory committee

1.3. ARC referral form developed by the research team in collaboration with advisory committee

2. Enhance equitable utilization of CR

2.1. A lay Patient Navigator, trained by the research team, provides navigation services to participating primary care patients

- The navigator is a lay person with strong interpersonal and communication skills. No clinical background.
- The navigator training program developed by the research team totals approximately 25 hours with 12 online modules and 5 face-to-face sessions. Navigators were trained on cultural competency, the active offer of French language services, motivational interviewing, and advocacy and empowerment.
- Professional development and mentorship through regular encounters with other community navigators

C. HOW

1. Enhance recommendations to CR

    1.1. Face-to-face orientation on community resources with PCPs at each participating practice (group session)

    1.2. Promotional material displayed in the practice waiting room and patient examining rooms (where feasible)

    1.3. Face-to-face training (group) and instructional video (online) on recommendation process

2. Enhance equitable utilization of CR

- Individual encounters with patients face-to-face or by telephone

D. WHERE

1. Enhance recommendations to CR

    1.1. Orientation to community resources will occur at the practice site

    1.2. Promotional material will be displayed in practice waiting rooms

    1.3. A referral form will be incorporated into routine practice (.e.g., via EMR or paper copies in patient examining rooms)

2. Enhance equitable utilization of CR

    2.1. Navigator services will be provided face-to-face (at the patient's practice or in a public space in the community) or by telephone

E. WHEN and HOW MUCH

1. Enhance recommendations to CR

    1.1. Orientation to community resources will be given to all participating providers once prior to patient recruitment.

    1.2. Promotional material will be displayed in practice waiting rooms prior to patient

recruitment, and can be adapted throughout the study.

1.3. Referral forms will be introduced in the practice at the beginning of the study. The referral process (e.g., content of the form; integration in routine activities) can change throughout the study.

2. Enhance equitable utilization of CR

2.1. Navigator services will be provided to patients up until 3 months after the end of the recruitment period.

F. TAILORING

1. Enhance recommendations to CR

1.1. Orientation to community resources will be tailored to patient priority needs identified by PCPs

1.2. Promotional material can be adapted throughout the study according to feedback from PCPs.

1.3. The referral process (e.g., content of the form; integration in routine activities) can be adapted based on PCP needs and preferences. Modifications can be made throughout the study period.

2. Enhance equitable utilization of CR

2.1. Navigator services will be offered in person or by telephone, at participating practice sites or in public locations (e.g., local CHC). Services will be personalized based on individual patients' needs and preferences.
