# Supplementary material for: Access to Resources in the Community Through Navigation: Protocol for a Mixed-Methods Feasibility Study
Source: JMIR Res Protoc. 2019 Jan 24;8(1):e11022. doi: 10.2196/11022 (PMC6365876; doi:10.2196/11022)
Supplement: Multimedia Appendix 4 [file resprot_v8i1e11022_app4.pdf]

Study on Access to Resources in the Community
Étude sur l'Accès aux ressources communautaires

uOttawa · Institut de recherche Bruyère Research Institute · The Ottawa Hospital / L'Hôpital d'Ottawa · CIHR IRSC · Ontario SPOR Support Unit · Savoir Montfort

AVAILABLE RESOURCES/RESSOURCES DISPONIBLES

7, 000 + services in your community!
7, 000 + services dans votre communauté!

- **Mental Health** – Santé mentale
- **Parenting support** – Soutien parental
- **Seniors' Health** – Santé des aînés
- **Falls prevention** – Prévention des chutes
- **Pain management** – Gestion de la douleur
- **Financial assistance** – Assistance financière
- **Caregiver support** – Soutien aux proches aidants

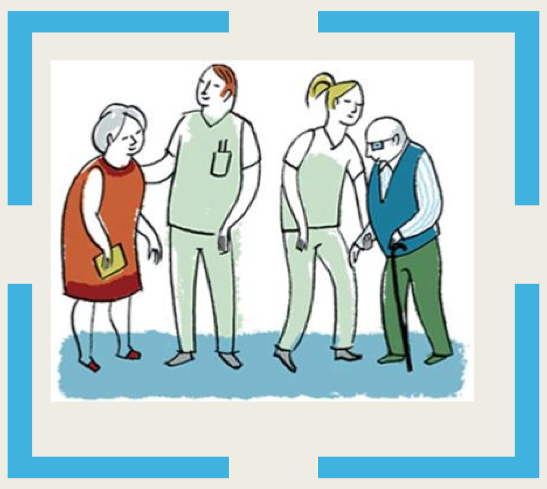
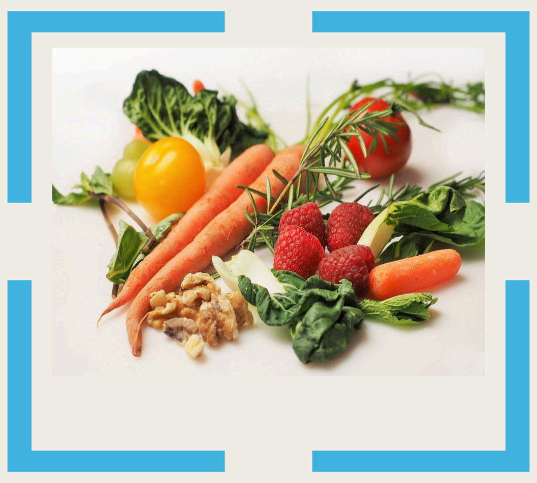
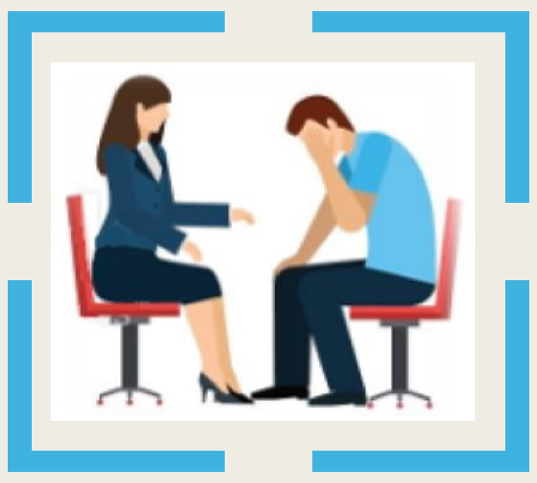
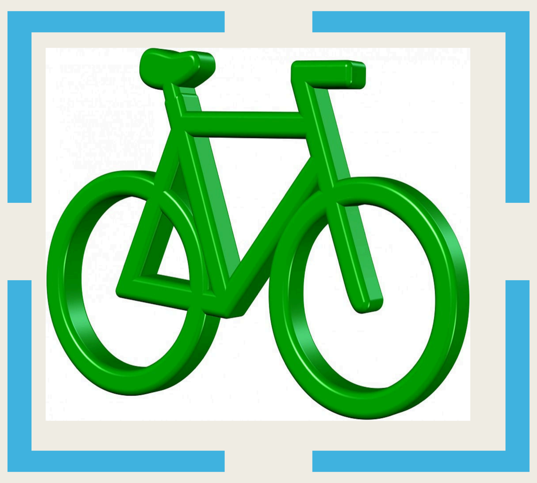

- **Foot care** – Soin des pieds
- **Quitting smoking** – Arrêter de fumer
- **Healthy eating** – Manger sainement
- **Social assistance** – Assistance sociale
- **Physical Activity** – Activité physique
- **Diabetes education** – Éducation sur le diabète
- **Addiction services** – Services de toxicomanie

HOW CAN THE ARC STUDY HELP YOU...
COMMENT L'ÉTUDE ARC PEUT VOUS AIDER ...

My Health Care
Referral

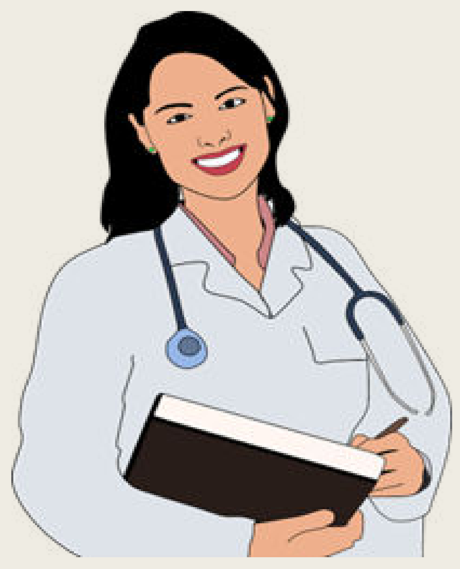

Ma pratique
Renvoi

Navigation
Support

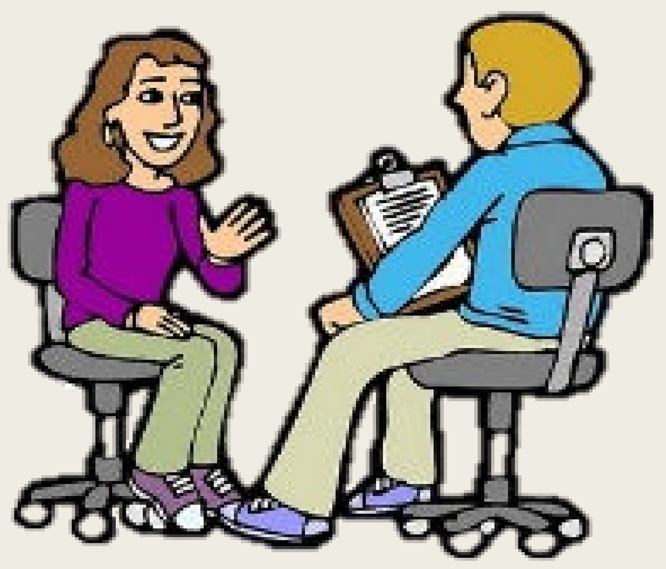

Navigation
Soutien

Community Service
Access the service!

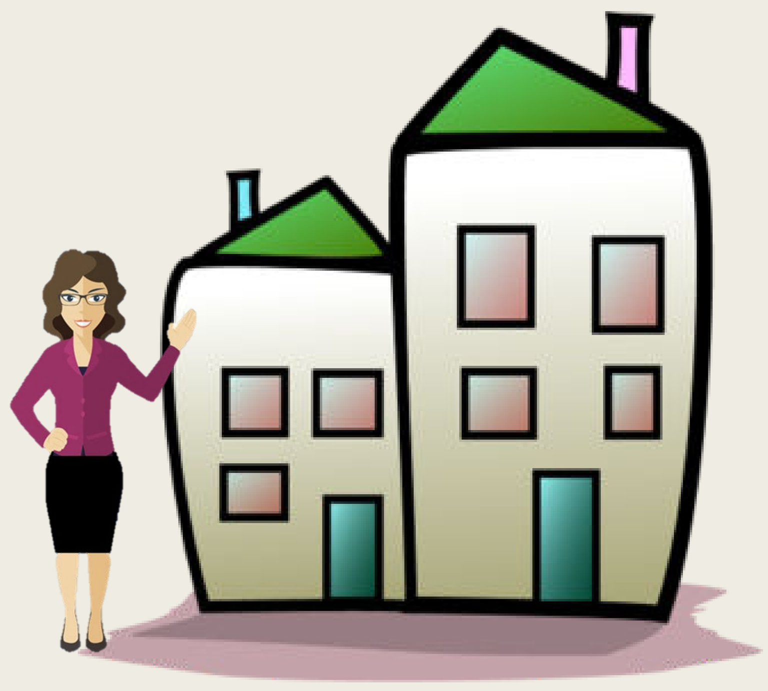

Service communautaire
Accéder au service!

Talk to your health care provider about the ARC study!
Parlez à votre fournisseur de soins de santé au sujet de l'étude ARC!

This study was approved by: Ottawa Health Science Network Research Ethics Board (#20160914-01H), Bruyere Continuing Care Research Ethics Board (#M16-16-055), l'Hôpital Montfort Research Ethics Board (#SD-DP-27-02-17), University of Ottawa Research Ethics Board (# A05-17-04)

Cette étude a été approuvée par le comité d'éthique de la recherche du Réseau de science de la santé d'Ottawa (#20160914-01H), le comité d'éthique en matière de recherche de Soins Continus Bruyère (#M16-16-055), le comité d'éthique de la recherche de l'Hôpital Montfort (#SD-DP-27-02-17), et le comité d'éthique et d'intégrité de la recherche Université d'Ottawa (# A05-17-04). [October 27th, 2017]
