# Supplementary material for: Access to Resources in the Community Through Navigation: Protocol for a Mixed-Methods Feasibility Study
Source: JMIR Res Protoc. 2019 Jan 24;8(1):e11022. doi: 10.2196/11022 (PMC6365876; doi:10.2196/11022)
Supplement: Multimedia Appendix 6 [file resprot_v8i1e11022_app6.pdf]

ARC Community Resource Referral Form

ARC
Access to Resources in the Community
Accès aux ressources communautaires

ARC Research Team Contact information:
Tel: 613-562-6262 ext. 2920 **Fax: 613-782-2777**

☐ The patient agrees to be contacted by the researcher team. If yes, complete shaded boxes

**Name:** ______________________________	**Telephone #:** (__ __ __) __ __ __ - __ __ __ __<br>**Best time:** ☐ **AM** ☐ **PM** ☐ **Evenings** ☐ **Weekends**<br>☐ **Other** ______________________________

Please select one option:

☐ Able to communicate with research team in French or English
Preferred language for contact:
☐ French ☐ English

☐ Requires interpretation services *please specify:* ____________________

☐ Requires support from parent or proxy. *Proxy Contact Information:*

Name: __________________________ Telephone #: ______________________

☐ The patient was provided with the study information package and the research team's contact information.

☐ **Male** ☐ **Female** ☐ **Other**	**Age:** __________ **years**

The following programs are recommended / of interest:

☐ Chronic pain	☐ Diabetes education	☐ Mental health
☐ Physical activity	☐ Stop smoking	☐ Addiction
☐ Healthy eating	☐ Parenting and family support	☐ Self-management

☐ Other (specify):

Additional Comments

Referring Provider's name (Please Print): ______________________

Signature: _____________________ **Date: 201_/_ _/_ _**
Year / Month / Day
